# From Cell‐Free Transcriptomes to Single‐Cell Landscapes: Biomarker Discovery and Originating Cell Alteration Analysis via Graph Matrix Factorization

**DOI:** 10.1002/advs.74814

**Published:** 2026-03-12

**Authors:** Wenxiang Zhang, Wenjing Zhang, Hang Wei, Shiyan Liu, Junliang Shang, Weijie Gong, Hanwen Cheng, Xiujuan Lei, Yuhui Kou, Baoguo Jiang

**Affiliations:** ^1^ Shenzhen Clinical Research Center for Trauma treatment Shenzhen University General Hospital Shenzhen University Shenzhen China; ^2^ Guangdong Key Laboratory For Biomedical Measurements and Ultrasound Imaging National‐Regional Key Technology Engineering Laboratory For Medical Ultrasound School of Biomedical Engineering Shenzhen University Medical School Shenzhen China; ^3^ National Center for Trauma Medicine Beijing China; ^4^ School of Computer Science and Technology Xidian University Xi'an Shaanxi China; ^5^ School of Computer Science Qufu Normal University Rizhao China; ^6^ Department of Family Medicine Shenzhen University Medical School Shenzhen Guangdong China; ^7^ Department of Orthopedics and Trauma Peking University People's Hospital Beijing China; ^8^ School of Artificial Intelligence and Computer Science Shaanxi Normal University Xi'an China

**Keywords:** cfRNA biomarker identification, cfRNA originating cells, clinical sample diagnostic classification, graph matrix factorization

## Abstract

Characterizing the cellular origin and disease‐driven dynamics of cfRNA is essential for integrating cfRNA profiling into clinical workflows and precision‐medicine strategies. Most cfRNA studies are restricted to bulk‐level analyses, which preclude detailed analysis of alterations in the cellular origins of cfRNA. Single‐cell RNA sequencing reveals cellular heterogeneity and communication, but its application to cfRNA is limited by diverse cellular origins, leaving a critical gap in understanding functional alterations in cfRNA biomarker‐originating cells. In this work, we propose CellFreeGMF, a tool designed to enable diagnosis classification of clinical samples, identify cfRNA biomarkers, and analyze the alterations in their originating cells based on graph matrix factorization. Furthermore, by utilizing cell–cell communication analysis, CellFreeGMF investigates the functional alterations occurring in the cfRNA originating cells under disease conditions. We validate CellFreeGMF on diverse cell‐free RNA transcriptome clinical datasets. In the case of pancreatic ductal adenocarcinoma (PDAC), CellFreeGMF not only identified cfRNA biomarkers but also traced their cellular origins to myeloid and T‐cell populations. Further analysis revealed significant transcriptomic differences in these cell populations between disease and normal groups. Our user‐friendly CellFreeGMF toolkit (https://cellfreegmf.readthedocs.io/) enables identifying cfRNA biomarkers and elucidating pathophysiological changes in their originating cells.

## Graphical abstract

CellFreeGMF traces plasma cfRNA to likely originating cell types by integrating single‐cell atlases with graph‐regularized matrix factorization. The method decomposes cfRNA profiles into sample–cell contributions to reconstruct pseudo single‐cell expression. This originating‐cell analysis suggests which cell types may contribute to disease‐associated signals and supports exploratory mapping of altered cell–cell communication patterns from liquid biopsy data.

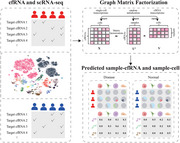

## 1 Introduction

Cell‐free RNAs (cfRNAs) are extracellular RNA fragments present in body fluids, released into the circulation through cellular metabolism and apoptosis from various tissues [1]. They comprise a diverse repertoire of RNA species, including long RNAs (e.g., mRNAs, lncRNAs, and circRNAs), as well as small RNAs (e.g., miRNAs, piRNAs, tsRNAs, and rsRNAs) [2, 3]. Importantly, cfRNA profiles reflect tissue‐specific alterations in gene expression, intercellular signaling, and the extent of cell death across different organs [4].

Unlike conventional intracellular RNAs, cell‐free RNAs (cfRNAs) are readily accessible through minimally invasive approaches, making them an attractive target for liquid biopsy with broad clinical applications. In a comprehensive transcriptome‐wide study, Larson et al. demonstrated cfRNAs as promising biomarkers for early cancer detection, enabling accurate prediction of tumor tissue of origin and classification of cancer subtypes, thereby advancing the clinical utility of liquid biopsy in oncology [4]. Bao et al. developed cfPeak, a computational framework that identifies recurrently protected cfRNA peaks overlapping with protein‐binding and vesicle‐sorting sites, and demonstrated its utility in cancer detection [5]. In addition, cfRNAs are also implicated in the prediction, diagnosis, and prognosis of various diseases, including tuberculosis [6], preeclampsia [7, 8], central nervous system diseases [9], gastrointestinal cancer [10], and pancreatic ductal adenocarcinoma [11, 12].

Despite these advances, most cfRNA‐based studies remain limited to bulk‐level analyses, which lack the resolution to determine the cellular origins of cfRNAs and provide little insight into their functional roles in disease biology [6, 7, 8, 9, 10, 11, 12, 13]. Single‐cell RNA sequencing enables high‐resolution analysis of cellular heterogeneity and intercellular communication [14, 15, 16]. With the establishment of large‐scale single‐cell reference atlases such as the Tabula Sapiens database, comprehensive multi‐organ single‐cell transcriptomic landscapes are now available, providing valuable resources for exploring disease mechanisms [17]. While single‐cell RNA sequencing has revolutionized our understanding of cellular heterogeneity and intercellular communication [18], its direct application to cfRNA remains challenging. Unlike cellular transcriptomes, cfRNA molecules are fragmented and released by diverse cell types across the human body [7, 13, 19], making it challenging to characterize the functional alterations in the cfRNA biomarker originating cells [13]. This limitation highlights a critical gap between cfRNA analysis and the mechanistic insights achievable with single‐cell approaches.

To bridge this gap, we developed CellFreeGMF, an open‐source Python package (https://github.com/zwx94/CellFreeGMF) to identify cfRNA biomarkers and analyze the functional alterations in their originating cells. CellFreeGMF not only enables diagnostic classification of disease samples and identification of cfRNA biomarkers but also elucidates the functional alterations of cfRNA originating cells based on cell‐free transcriptome and single‐cell sequencing data. CellFreeGMF first employs machine learning algorithms and differential expression analysis to perform sample classification and cfRNA biomarker identification. Subsequently, CellFreeGMF integrates single‐cell reference data with cfRNA transcriptomes to simulate two sample‐cell association matrices, including disease samples to cell types and normal samples to cell types. It also predicts the corresponding single‐cell expression profiles for both disease and normal groups. This is achieved through a graph‐regularized matrix factorization framework based on the transcriptomic data of identified cfRNA biomarkers. The inferred sample–cell association matrices reveal cfRNA origins and functional alterations in the originating cells, while the reconstructed single‐cell profiles capture transcriptional changes between disease and normal conditions. To highlight the applicability of CellFreeGMF, we applied it to three publicly available datasets, including tuberculosis [6], preeclampsia [7], and pancreatic ductal adenocarcinoma [11].

## 2 Results

### 2.1 Overview of CellFreeGMF

**FIGURE 1:**
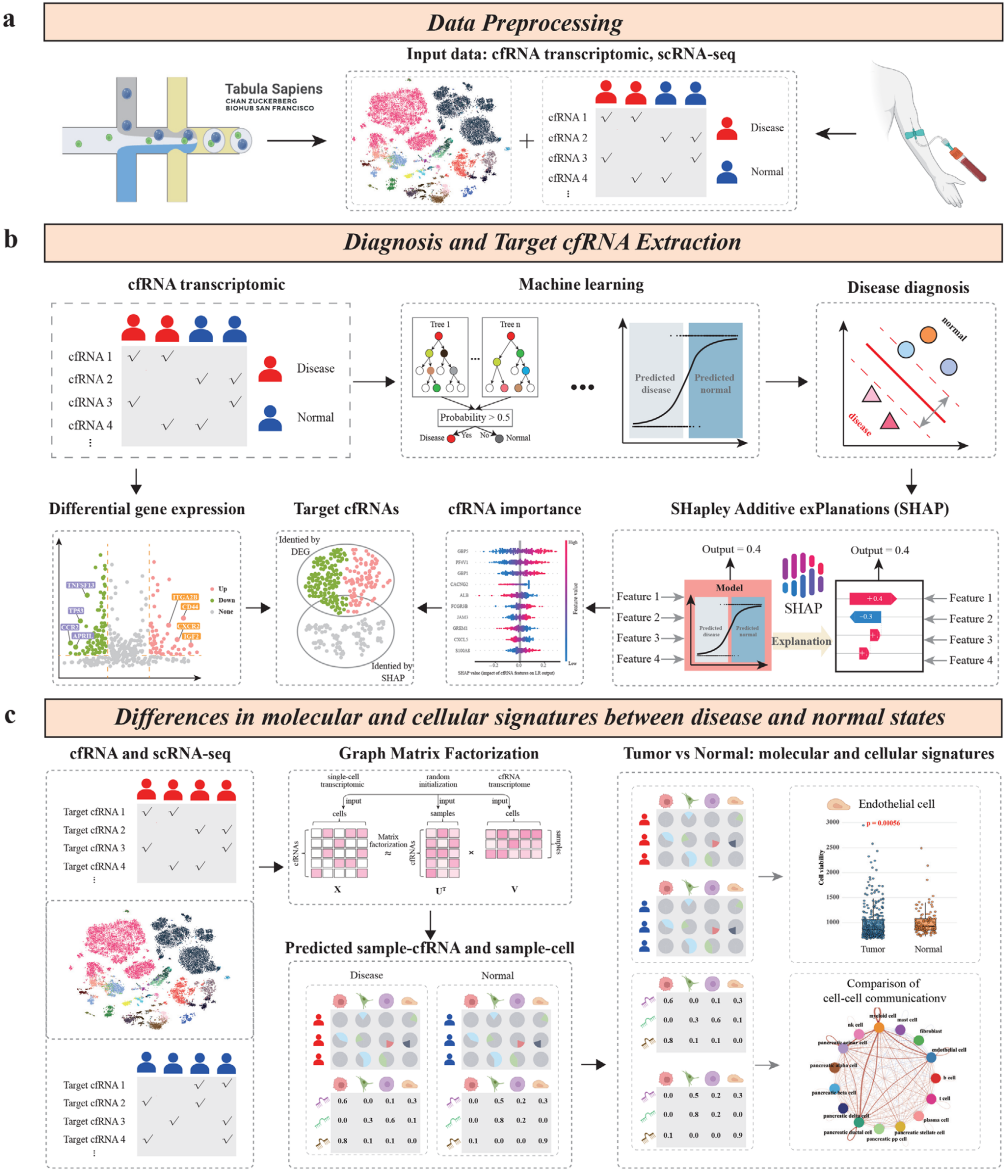
Workflow of CellFreeGMF. (a) Extraction of cell‐free and single‐cell transcriptomic data. (b) Disease classification and cfRNA biomarker extraction. (c) Differences in molecular and cellular signatures between disease and normal states.

CellFreeGMF requires both cell‐free and single‐cell transcriptomic data as user input to identify cfRNA biomarkers and uncover significant alterations in their origin cells between disease and normal control groups (Figure 1a). Upon receiving these inputs, CellFreeGMF performs two key operations to achieve its objectives:

The discovery of cfRNA biomarkers differentially expressed in disease vs. normal samples is crucial for downstream deconvolution and interpretation. This selection step ensures that only biologically meaningful transcripts are used in origin tracing and cell‐state comparison. To reliably identify disease‐associated cfRNA biomarkers, we employed two complementary strategies (Figure 1b). We first performed differential expression analysis to identify target cfRNAs with significant changes between disease and normal samples. Second, we applied a machine learning–based classification framework using cfRNA transcriptomic profiles to distinguish disease from normal samples. To interpret the model and extract informative features, we utilized SHapley Additive exPlanations (SHAP) [20], identifying cfRNA biomarkers that contributed most to the classification performance. These complementary strategies leverage both statistical inference and model‐driven feature importance, enabling the identification of cfRNA biomarkers that are not only differentially expressed but also diagnostically informative and biologically relevant.

Tracing the functional alterations in the originating cells of disease‐associated cfRNA biomarkers is crucial for understanding the cellular context and biological mechanisms underlying disease‐related transcriptomic changes. To achieve this, we integrated single‐cell RNA sequencing data with cfRNA transcriptomic profiles, focusing exclusively on the disease‐relevant cfRNA biomarkers identified in the first step. These integrated data were then fed into a graph‐regularized matrix factorization framework, which decomposes the data into two key matrices: a sample–cell association matrix and a single‐cell expression matrix (Figure 1c). The sample–cell matrix reveals the identification of cell types showing significant differences between disease and normal groups. Moreover, the single‐cell expression matrix reflects the expression patterns of disease‐related cfRNAs within individual cell types, facilitating the analysis of cell–cell communication changes between disease and normal conditions.

CellFreeGMF provides a comprehensive and generalizable analytical framework encompassing the entire pipeline of cfRNA‐based disease analysis—from disease classification and biomarker identification to functional alterations in cfRNA originating cells. By integrating transcriptomic profiles with single‐cell references through graph‐regularized matrix factorization, CellFreeGMF enhances the interpretability of cfRNA data and uncovers both molecular and cellular signatures of disease. This approach is broadly applicable across diverse diseases, offering a scalable solution for non‐invasive transcriptomic analysis.

### 2.2 cfRNA‐Based Disease Diagnostic Classification and Biomarker Identification

**FIGURE 2:**
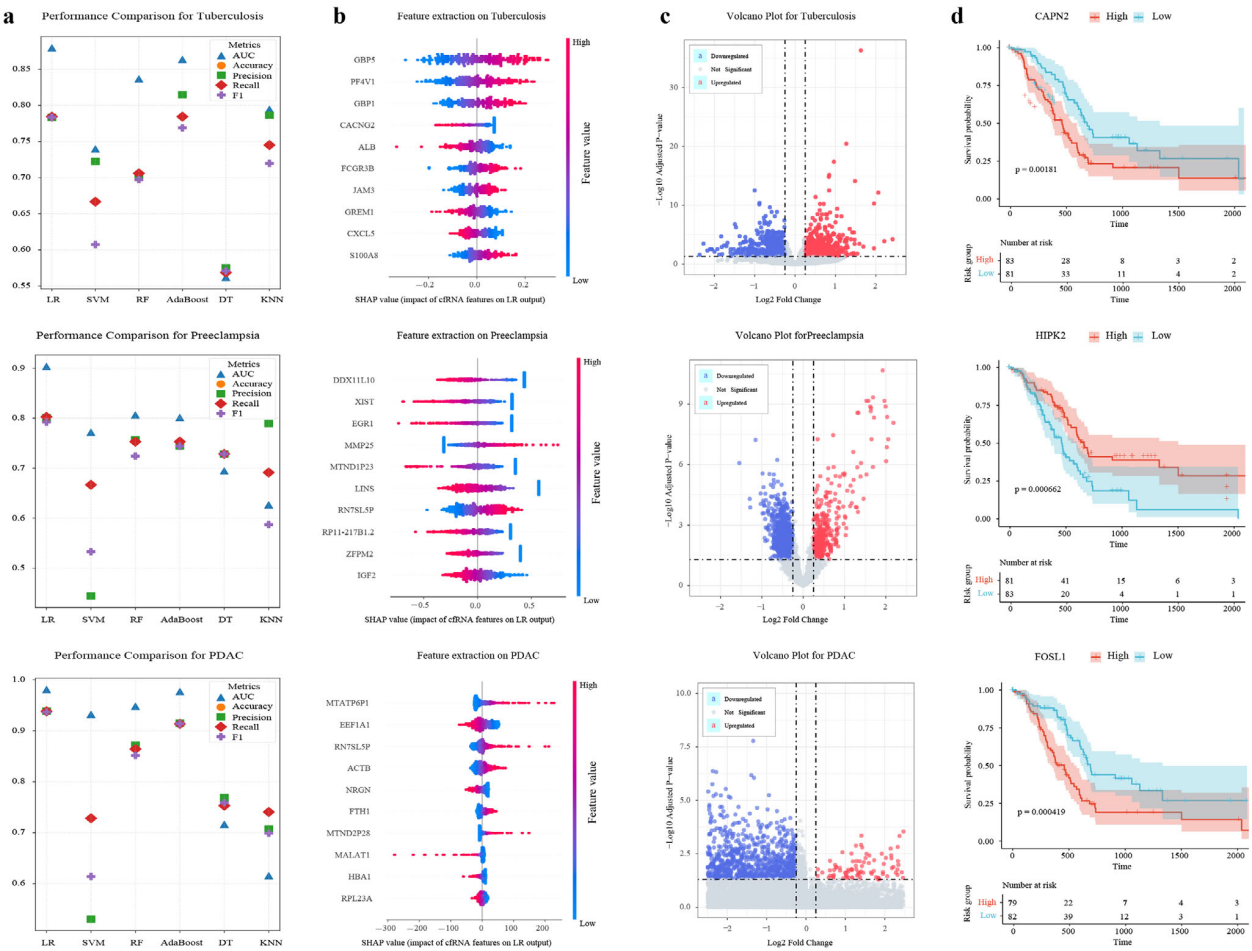
Diagnostic classification and cfRNA biomarker identification based on cell‐free Transcriptomes. (a) Performance evaluation of six machine learning algorithms for diagnostic classification based on cfRNA transcriptome data. (b) The top 10 cfRNA features driving the LR model's diagnostic classification for tuberculosis, preeclampsia, and PDAC. (c) Volcano plots showing differentially expressed genes in tuberculosis, preeclampsia, and PDAC. Red indicates significantly upregulated genes, while blue represents downregulated genes. (d) Kaplan‐Meier analysis of PDAC survival stratified by plasma cfRNA biomarker levels (high vs. low, median split) in TCGA‐PAAD (*n* = 178 patients; log‐rank test).

To achieve a more comprehensive identification of disease‐associated cfRNAs, CellFreeGMF first employed six machine learning algorithms to classify samples based on cfRNA transcriptomic data. The performances of these algorithms, including logistic regression (LR), support vector machine (SVM), random forest (RF), AdaBoost, decision tree (DT), and k‐nearest neighbor (KNN), are shown in Figure 2a. Among these, logistic regression exhibited the highest classification accuracy, indicating its suitability for cfRNA‐based sample classification. Subsequently, the trained LR model was interpreted using SHAP to calculate the importance of each cfRNA feature in the classification process. The resulting feature importance values are shown in Figure 2b and Table S1. cfRNAs ranked within the top 10% in terms of importance were selected as candidate disease‐associated cfRNAs.

In parallel, differential expression analysis was also performed (Figure 2c; Table S2). The cfRNA biomarkers identified by both approaches were integrated, and their overlaps and differences were illustrated in Figure S1. Biomarkers exclusively identified through differential expression analysis exhibited strong statistical significance (e.g., Log2Fold Change indicating expression differences and *p*‐values reflecting statistical confidence). However, the biological validity of biomarkers identified exclusively by SHAP required further evaluation. To this end, we analyzed their association with patient survival using pancreatic ductal adenocarcinoma data from The Cancer Genome Atlas (TCGA). As shown in Figure 2d and Table S3, many SHAP‐identified biomarkers were significantly correlated with patient survival. Moreover, many of these biomarkers were previously implicated in PDAC pathogenesis. For instance, CAPN2 was identified as a PDAC‐enriched intracellular protease that enables the design of CAPN2‐responsive mesoporous silica nanoparticles, which enhance targeted paclitaxel delivery and effectively suppress tumor growth in pancreatic ductal adenocarcinoma [21]. HIPK2 promoted KRAS‐driven pancreatic tumorigenesis, and its inactivation suppressed ERK signaling, reduced PanIN formation, and attenuated PDAC development [22]. FOSL1, a YAP/TEAD‐regulated gene and component of AP‐1, was associated with poor survival in pancreatic ductal adenocarcinoma, highlighting its role in promoting PDAC aggressiveness through the Yes‐associated protein signaling network [23]. Therefore, this integrative strategy, combining machine learning–based feature interpretation with differential expression analysis, enables robust and biologically meaningful identification of disease‐associated cfRNA biomarkers, thereby enhancing both diagnostic accuracy and mechanistic insight.

### 2.3 Compare the Predicted Sample‐cfRNA between the Disease Group and the Normal Group

**FIGURE 3:**
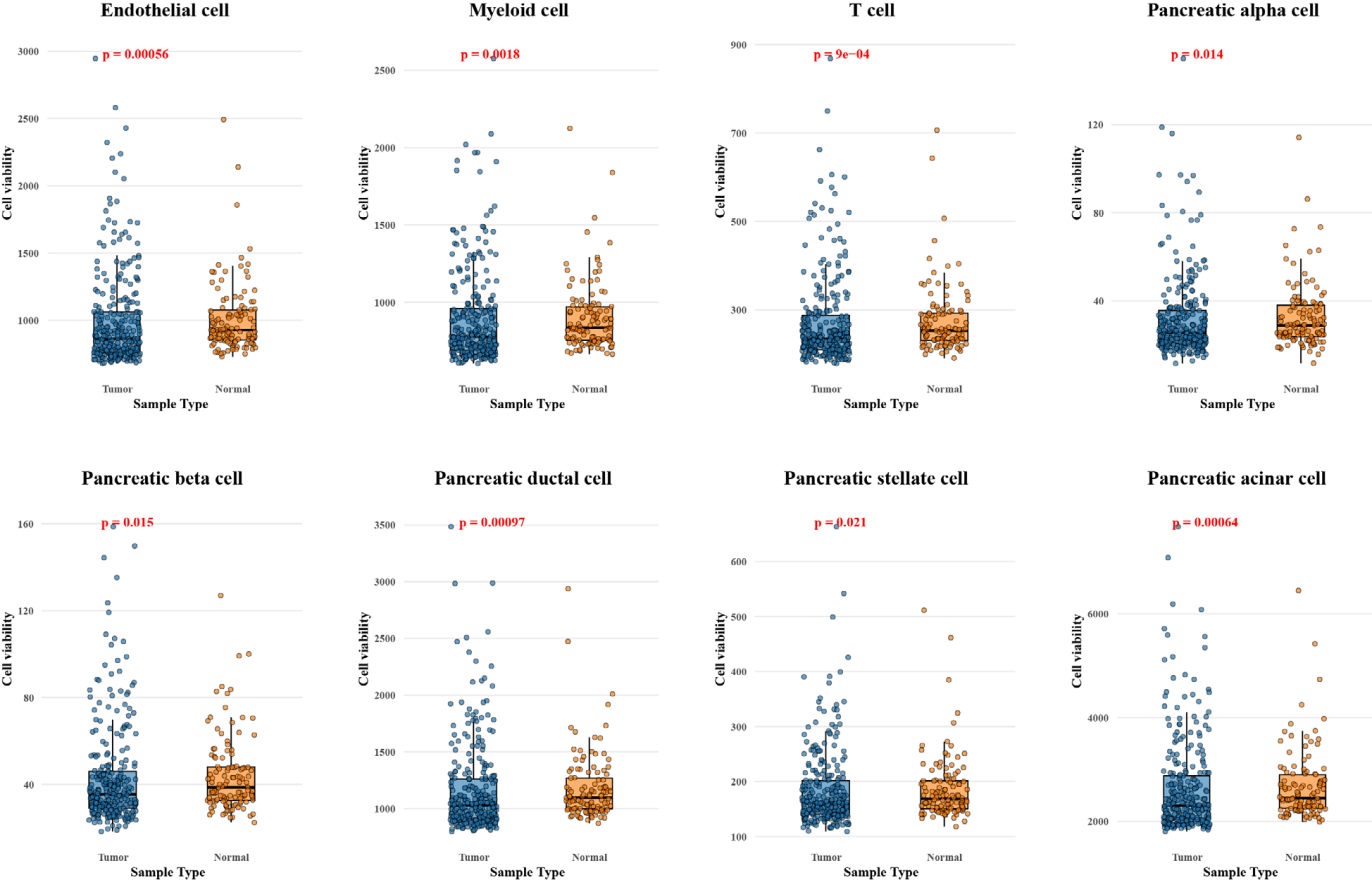
Cell types significantly differing between PDAC and normal group. Two‐sided Wilcoxon rank‐sum tests were performed for each cell type to compare Normal vs. Tumor samples. Significant results (*p* < 0.05) are shown.

CellFreeGMF employed a single‐cell reference dataset to construct association matrices that quantify relationships between disease/normal samples and specific cell types (see “Experimental Section”). Comparing these matrices enabled the systematic identification of differences in cell–sample associations, thereby providing insights into cell–type–specific alterations linked to disease. The analysis identified eight cell types with significant differences in sample–cell associations between PDAC and normal conditions, notably including pancreas‐associated cells, myeloid cells, and T cells (see Figure 3; Table S4). There is already substantial evidence linking pancreas‐associated cells to pancreatic ductal adenocarcinoma. For instance, Pancreatic stellate cells support pancreatic ductal adenocarcinoma progression by secreting alanine through autophagy, which fuels tumor cell metabolism and promotes cancer growth [24]. Pancreatic acinar cells can transdifferentiate into pancreatic ductal cells under KRAS‐driven PDAC development, with TAK1 playing a critical role in determining acinar cell fate toward either cancer progression or cell death [25].

In addition to the five pancreatic cell types implicated in PDAC, our analysis identified three other cell populations—endothelial cells, myeloid cells, and T cells—that also exhibit significant associations with the disease (see Figure 3). Endothelial cell enrichment is observed in immune‐hot PDAC tumors, where it is strongly associated with cytotoxic immune cell infiltration, suggesting a potential role in modulating the tumor immune microenvironment [26]. In PDAC, modulation of T cell subsets to enhance antitumor activity and reduction of immunosuppressive myeloid cells can significantly improve antitumor immunity and survival. Combined targeting of T cell checkpoints (41BB, LAG3) and myeloid cell CXCR1/2 yields durable complete responses, suggesting a promising therapeutic approach for this otherwise non‐immunogenic cancer [27]. Our computational analysis identified cell types showing significant differences between PDAC and normal sample–cell association matrices, and literature evidence from mechanistic studies further substantiated the relevance of these cells to PDAC pathogenesis. The concordance between our predictions and experimentally validated findings supports the accuracy of our approach. These results demonstrate that our method can not only detect disease‐associated cellular alterations with high confidence but also provide biologically meaningful insights into the cellular landscape of PDAC, thereby offering a valuable framework for integrating computational predictions with experimental evidence in cancer research.

### 2.4 Compare the Cell–Cell Communication Difference between the PDAC Group and the Normal Group

**FIGURE 4:**
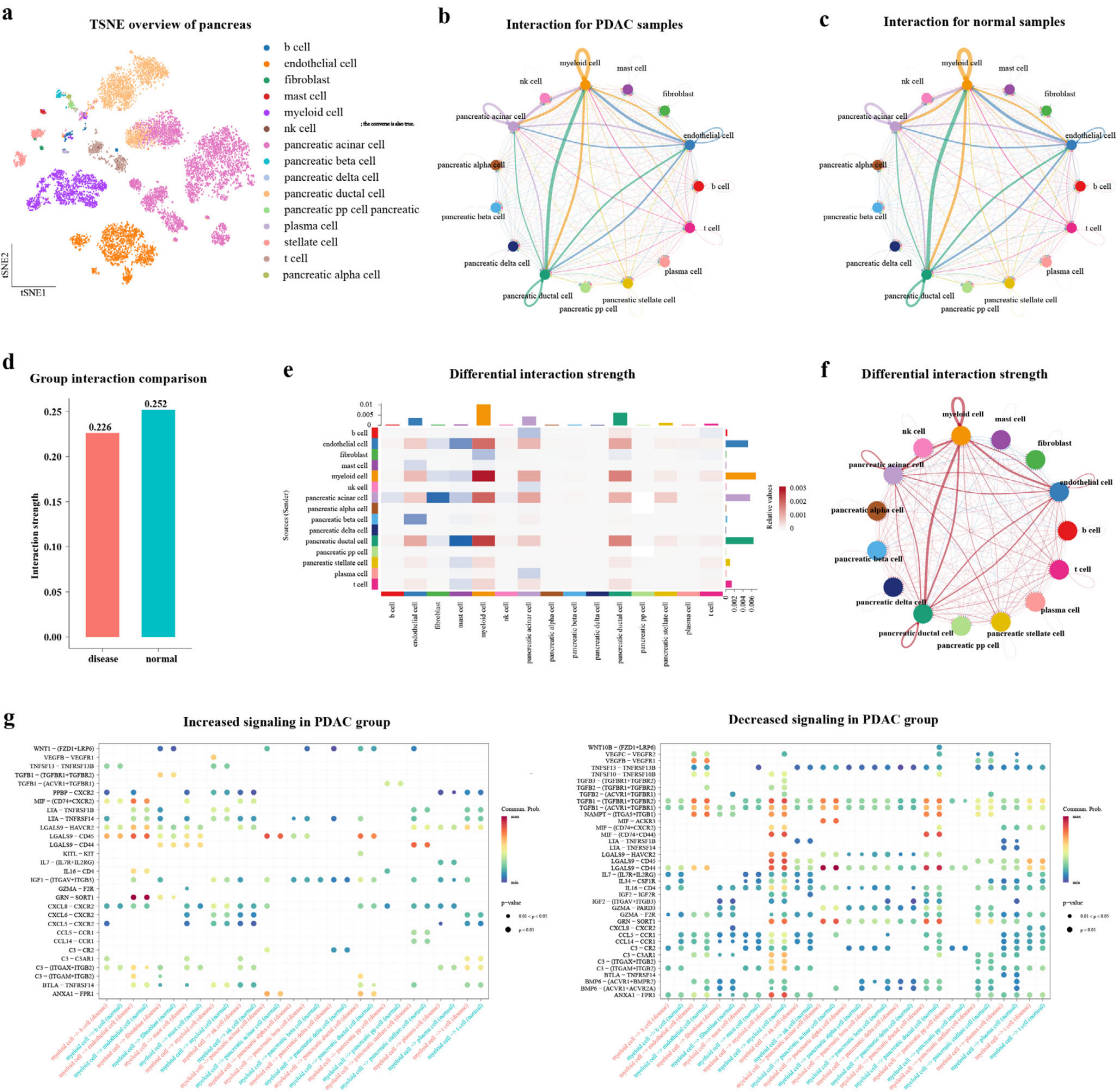
Overview of cell–cell communication differences between PDAC and normal groups. (a) The t‐distributed stochastic neighbor embedding (t‐SNE) plot of the pancreatic single‐cell reference dataset. (b,c) circle plots representing cell–cell communication networks in disease and normal groups, with edge widths scaled to interaction strength, respectively. (d) Comparison of cell–cell interaction strengths between PDAC and normal groups based on communication weight. (e) Heatmap illustrating the differential strength of cell–cell communication interactions between PDAC and normal group, measured by signaling weight. (f) Differential intercellular communication network showing the scaled differences in interaction strength between PDAC and normal groups. g and h, Bubble plots showing significantly increased and decreased ligand–receptor interactions between the indicated myeloid cell and target cell types in the PDAC group compared with the normal groups.

CellFreeGMF employs graph matrix factorization to predict single‐cell transcriptomic profiles of disease and normal groups by integrating cell‐free transcriptome data with a pancreatic single‐cell reference dataset (see “Experimental Section” and Figure 4a). By comparing cell–cell communication patterns between predicted single‐cell transcriptomic profiles of PDAC and normal groups, including differences in interaction strength, signaling pathways, and ligand–receptor pairs, we aimed to uncover disease‐specific intercellular signaling alterations. This approach provided mechanistic insights into the cellular crosstalk underlying PDAC pathogenesis and revealed potential therapeutic targets. The predicted cell–cell communication interactions for PDAC and normal samples, as well as their differences, were presented in Figure 4b–f. Notably, the most pronounced differences in interaction strength between PDAC and normal samples were observed in endothelial cells, myeloid cells, pancreatic acinar cells, and pancreatic ductal cells. These findings are consistent with the results shown in Figure 3.

We examined significantly altered ligand–receptor interactions between the identified communication‐differing cells and their target cell types in PDAC vs. normal samples (see Figure 4g,h; Figures S2–S4). A substantial number of ligand–receptor pairs were detected exclusively in the PDAC group, and were absent in the normal group; the opposite was also observed. Notably, several of the absent ligand‐receptor pairs, such as CXCL5–CXCR2, VEGFB–VEGFR1, and CCL5–CCR1, have been previously reported to be associated with PDAC, supporting their potential relevance to disease progression and tumor–microenvironment interactions. Upregulation of CXCL5 promotes recruitment of myeloid‐derived suppressor cells and suppression of CD8^+^ T cells, effects that can be mitigated by combined CXCR2 and CCR2 inhibition, thereby restraining the accelerated progression of PDAC following stromal Col1 deletion [28]. Huang et al.’s findings indicate that the combined expression profile of VEGFRs (VEGFR1, 2, and 3) serves as a prognostic marker for PDAC, and that amiRNA‐mediated suppression of VEGFR expression represents a potential targeted therapeutic strategy by modulating epithelial‐mesenchymal transition [29]. In PDAC, PD‐L1 was a direct transcriptional target of cancer cell–derived FOXP3, and combined immunotherapy targeting PD‐L1 and CCL5 demonstrates therapeutic efficacy [30].

Pathway‐level analysis revealed that several signaling pathways were differentially regulated between the PDAC and normal groups. Notably, pathways such as APRIL (hsa04060, Cytokine–cytokine receptor interaction) exhibited significantly increased activity in PDAC (see Figure S5). APRIL predominantly mediates its biological effects through two specific ligand‐receptor interactions, namely TNFSF13‐TNFRSF17 and TNFSF13‐TNFRSF13B. Serum and lesion levels of TNFSF13 were significantly correlated with PDAC‐associated diabetes mellitus, suggesting a role for TNFSF13 in PDAC‐related inflammation [31]. Testoni et al. found that Elevated serum APRIL/TNFSF13 levels were associated with poor overall survival in patients with pancreatic ductal adenocarcinoma [32]. By integrating cell‐free transcriptome data with single‐cell references through graph matrix factorization, our approach enabled high‐resolution prediction of disease‐specific cellular communication networks in PDAC, uncovering clinically relevant ligand–receptor interactions and signaling pathways with potential diagnostic and therapeutic implications.

### 2.5 Application to Preeclampsia

**FIGURE 5:**
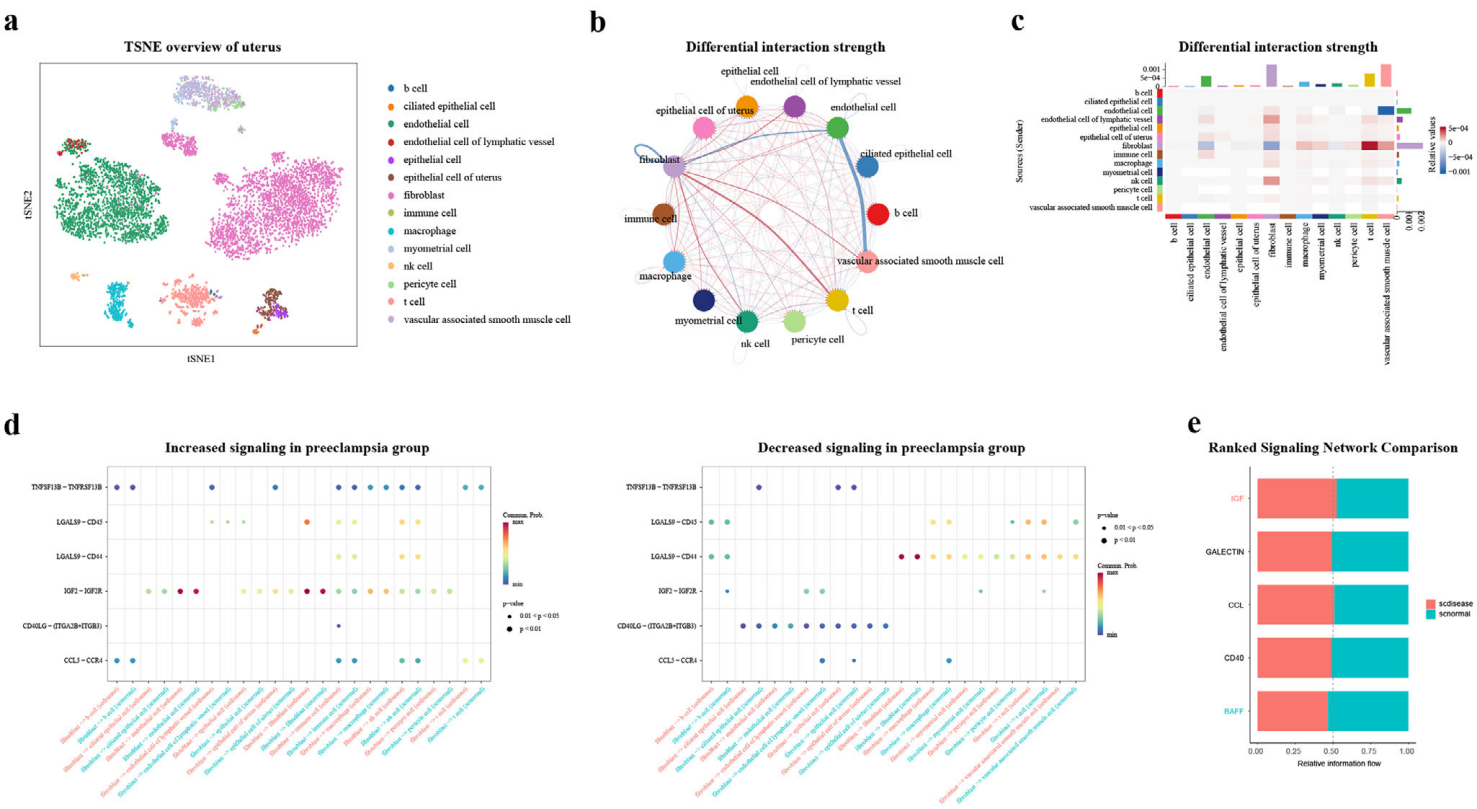
Cell–Cell communication analysis for preeclampsia. (a) The t‐distributed stochastic neighbor embedding (t‐SNE) plot of the uterus single‐cell reference dataset. (b) Differential intercellular communication network showing the scaled differences in interaction strength between preeclampsia and normal groups. (c) Heatmap illustrating the differential strength of cell–cell communication interactions between preeclampsia and normal groups, measured by signaling weight. (d) Bubble plots showing significantly increased and decreased ligand–receptor interactions between the indicated fibroblast cell and target cell types in the preeclampsia groups compared with the normal group. (e) Significant signaling pathways were ranked based on differences in the overall information flow within the inferred networks between preeclampsia and normal groups.

To rigorously evaluate the generalizability of CellFreeGMF beyond pancreatic ductal adenocarcinoma, we extended its application to preeclampsia and tuberculosis datasets. We first examined preeclampsia, a pregnancy‐specific complication marked by abnormal placental development, which remains a leading cause of maternal and perinatal morbidity and mortality worldwide [7, 8]. Based on a pancreatic single‐cell reference dataset and cell‐free transcriptomic data, CellFreeGMF employed graph matrix factorization to infer single‐cell transcriptomic profiles for both preeclampsia and normal groups (see “Experimental Section” and Figure 5a). The predicted cell–cell communication interactions for preeclampsia and normal samples, as well as their differences, were presented in Figure 5b–e. Striking alterations were predominantly observed in endothelial cell, fibroblast, t cell, and vascular‐associated smooth muscle cell, indicating that these cell types are highly likely to participate in the disease‐associated signaling landscape. To further substantiate this observation, we reviewed the literature and found substantial evidence supporting their involvement in preeclampsia. For example, Lip et al. found that endothelial cells in preeclampsia exhibit impaired proliferation, migration, and tube‐formation capacity, largely mediated by dysregulated plasma microRNAs [33].

In addition, we further analyzed the altered pathways and ligand–receptor interactions between the identified cells and their target cell types in preeclampsia compared with the normal group (Figure 5d,e; Figures S6–S8). Evidently, numerous ligand–receptor interactions were differed between the preeclampsia and normal groups, with several pairs such as LGALS9−CD44, IGF2−IGF2R, CD40LG−(ITGA2B+ITGB3). Notably, many of the cfRNAs involved in these differential ligand–receptor pairs have already been reported to be associated with preeclampsia [34, 35, 36]. For example, ITGB3 expression is markedly reduced in preeclamptic placentas, and its downregulation impairs trophoblast proliferation, migration, invasion, and adhesion, thereby contributing to disease progression [34].

### 2.6 Application to Tuberculosis

**FIGURE 6:**
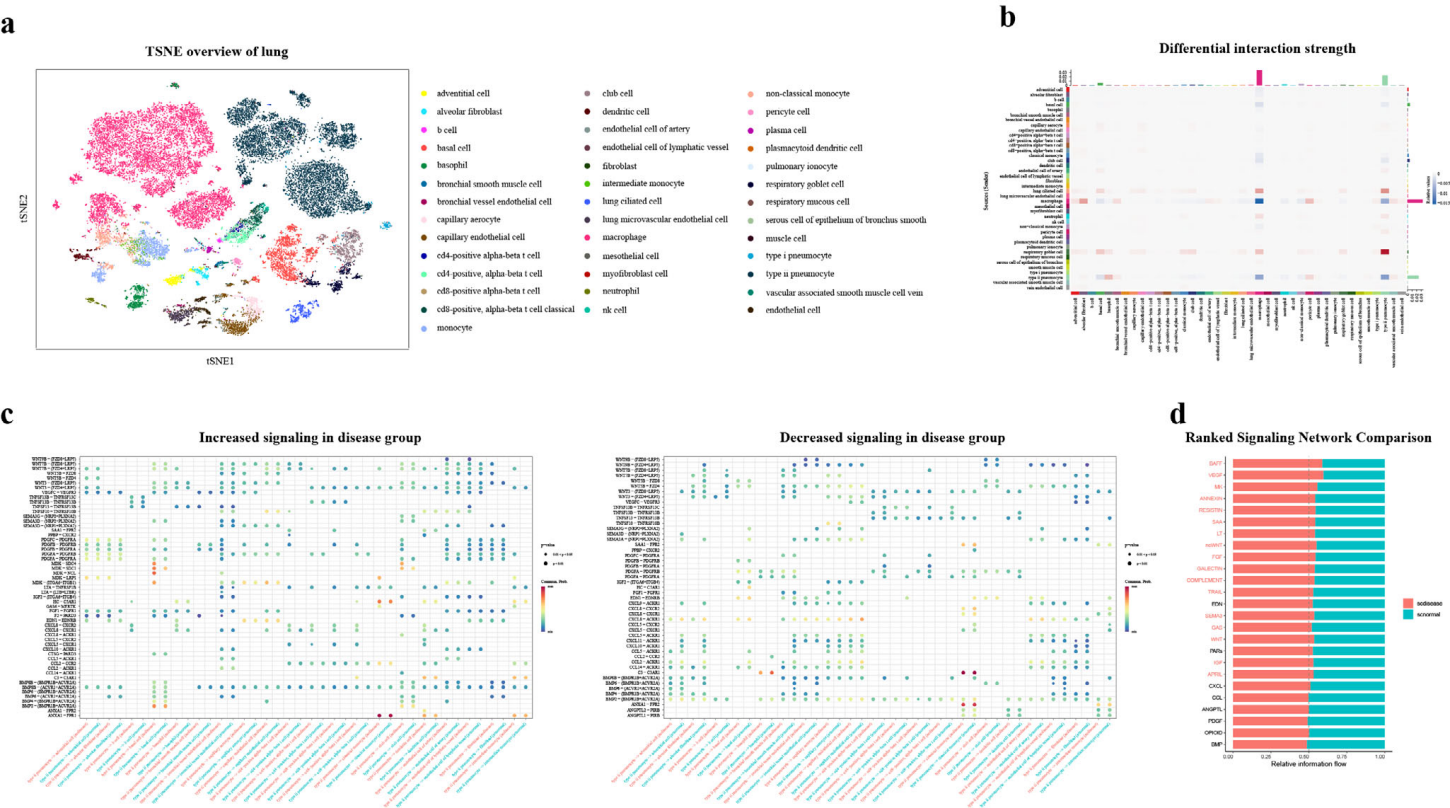
Cell–Cell communication analysis for tuberculosis. (a) The t‐distributed stochastic neighbor embedding (t‐SNE) plot of the lung single‐cell reference dataset. (b) Heatmap illustrating the differential strength of cell–cell communication interactions between tuberculosis and normal groups, measured by signaling weight. (c) Bubble plots showing significantly increased and decreased ligand–receptor interactions between the indicated type ii pneumocyte and target cell types in the tuberculosis groups compared with the normal group. (d) Significant signaling pathways were ranked based on differences in the overall information flow within the inferred networks between the tuberculosis and normal groups.

To further test the versatility of CellFreeGMF, we applied it to tuberculosis, a highly prevalent infectious disease that continues to cause considerable morbidity and mortality, particularly in low‐ and middle‐income countries [6, 37, 38]. Based on a lung single‐cell reference dataset and tuberculosis cfRNA transcriptomic data, CellFreeGMF inferred single‐cell profiles for tuberculosis and normal groups (see “Experimental Section” and Figure 6a). The predicted cell–cell communication differences were mainly concentrated in type ii pneumocytes, macrophage, club cell, and other cell types, as illustrated in Figure 6b. Numerous studies have demonstrated the relevance of these cell types to tuberculosis [39, 40]. For example, Tomioka et al. reported that type ii pneumocytes and macrophages serve as important host cells for Mycobacterium tuberculosis, providing intracellular niches that influence bacterial drug susceptibility [39]. Differential analysis of signaling pathways and ligand–receptor interactions identified numerous cfRNA biomarker candidates associated with tuberculosis, such as CXCL8, CCL2, CXCR1, and CXCR2 (see Figure 6b–d; Figures S9–S12). Notably, several of these altered ligand–receptor pairs have been previously implicated in tuberculosis pathogenesis. CXCL8 and CCL2 are strongly induced in human monocytes in response to the 30‐kDa antigen of Mycobacterium tuberculosis, implicating them in tuberculosis‐associated immune responses [41]. Together, these results demonstrated that CellFreeGMF robustly is generalizable across malignant, pregnancy‐related, and infectious diseases, enabling accurate reconstruction of disease‐specific single‐cell transcriptomic landscapes and uncovering critical cellular and molecular mechanisms.

## 3 Conclusion

In this study, we developed CellFreeGMF, a graph matrix factorization–based framework that integrates cfRNA transcriptomes with single‐cell reference data to infer disease‐specific single‐cell landscapes. By applying CellFreeGMF to pancreatic ductal adenocarcinoma, preeclampsia, and tuberculosis, we demonstrated its robust generalizability across malignant, pregnancy‐related, and infectious diseases. CellFreeGMF not only enabled the identification of cfRNA biomarkers and their cellular origins but also uncovered disease‐specific alterations in cell–cell communication and signaling pathways, providing mechanistic insights into disease progression.

Current cfRNA analyses mainly rely on bulk‐level differential expression, but this approach lacks the resolution needed to link cfRNA changes to their originating cells. Despite major advances in single‐cell analysis, including cell–cell communication inference, applying these methods to cfRNA is difficult since plasma‐derived RNA does not provide single‐cell resolution. CellFreeGMF bridges this gap by leveraging single‐cell reference atlases and graph matrix factorization to generate high‐resolution disease‐specific single‐cell profiles from cfRNA data. This integrative design allows the simultaneous biomarker identification, identification of functional alterations in disease‐relevant cell types.

Application of CellFreeGMF to PDAC revealed immune functional alterations, particularly in myeloid and T cell populations, consistent with prior studies on tumor microenvironmental remodeling [26, 27]. In PE, CellFreeGMF highlighted aberrant communication involving endothelial cells, fibroblasts, T cells, and vascular smooth muscle cells, aligning with known roles of endothelial dysfunction and abnormal placental vascular remodeling in disease pathogenesis [33, 34, 35, 36]. For TB, the model identified significant alterations in type II pneumocytes, macrophages, and club cells, which are well‐documented host niches for Mycobacterium tuberculosis and key regulators of immune defense [39, 40, 41]. These findings collectively underscore the capacity of CellFreeGMF to recapitulate biologically meaningful disease mechanisms from cfRNA perspective.

The major strength of CellFreeGMF lies in its ability to move beyond simple differential analysis of cfRNA data by providing mechanistic interpretability at the single‐cell level. By coupling cfRNA with single‐cell reference datasets, CellFreeGMF not only offers diagnostic capabilities but also enables hypothesis generation regarding cellular interactions and signaling pathways involved in disease. Strikingly, the patterns revealed by CellFreeGMF are concordant with established experimental findings across multiple diseases, lending strong support to both the validity of our approach and the robustness of cfRNA‐based single‐cell inference. To ensure broad accessibility, we have implemented CellFreeGMF as an open‐source Python package accompanied by an online user guide (https://github.com//zwx94//CellFreeGMF), thereby enabling the community to readily apply the framework for cfRNA‐based single‐cell analysis. Although CellFreeGMF shows strong performance and broad potential, it still has several limitations. First, the method depends on the quality and suitability of the single‐cell reference atlas; if the reference and cfRNA differ in tissue source, platform, batch, or disease context, the predicted results may be affected. Second, the pseudo–single‐cell expression profiles are inferred from plasma cfRNA, so they should be interpreted with caution. In future work, we will integrate multiple reference atlases, improve batch correction and uncertainty estimation, and validate key biomarkers and signaling findings in additional cohorts.

In conclusion, CellFreeGMF established a versatile framework for cfRNA‐based single‐cell inference and functional characterization, offering new opportunities to bridge liquid biopsy with single‐cell biology and advance precision medicine across diverse pathological conditions. To bridge liquid biopsy with single‐cell biology and advance precision medicine across diseases, the detailed installation and usage tutorials of CellFreeGMF are provided at https://cellfreegmf.readthedocs.io/. The CellFreeGMF code is openly available on GitHub (https://github.com/zwx94/CellFreeGMF) and distributed via PyPI (https://pypi.org/project/CellFreeGMF/).

## 4 Experimental Section

### 4.1 Data Preprocessing

CellFreeGMF required both cfRNA transcriptomic data and single‐cell transcriptomic reference data as inputs. To evaluate the generalizability of the framework, we employed three cfRNA datasets, including tuberculosis [6], preeclampsia [7], and pancreatic ductal adenocarcinoma [11]. For each disease, a corresponding single‐cell dataset was obtained from the Tabula Sapiens database [17], namely, lung, uterus, and pancreas. To ensure consistency between cfRNA and single‐cell references, redundant genes in the single‐cell datasets were removed, retaining only those that were also present in the cfRNA datasets. This preprocessing step ensured that each cfRNA feature was represented in the single‐cell reference, thereby facilitating accurate integration and downstream analysis.

### 4.2 Disease Diagnostic Classification and Biomarker Identification

For each disease dataset, samples were randomly split into a training set (80%) and a validation set (20%). To standardize cfRNA features, we first normalized cfRNA counts to CPM and then log‐transformed them (log2(CPM + 1)) prior to training the classifiers. All candidate classifiers (logistic regression, SVM, random forest, AdaBoost, decision tree, and k‐nearest neighbors) were trained on the same training split and evaluated on the same validation split to ensure a fair comparison. Model performance was reported on both training and validation sets using accuracy, precision, recall, F1‐score, and AUROC. The final classifier used for downstream biomarker identification was selected based on the overall validation performance, with logistic regression chosen as it achieved the best validation metrics in our benchmark. LR was implemented using *sklearn.linear_model.LogisticRegression* with *max_iter = 200*; all other hyperparameters were kept at scikit‐learn defaults. To identify cfRNA biomarkers, we applied SHAP method, which provides feature‐level interpretability by quantifying the contribution of each cfRNA to the classification outcome. cfRNAs with the highest SHAP importance values were designated as biomarkers for subsequent analyses.

### 4.3 Differential Expression Analysis

For differential expression analysis, DESeq2 method was used only for count‐based DE testing; limma method was used for log2(TPM) continuous expression. Differential expression analysis was performed separately for each disease to accommodate the data formats of the cfRNA transcriptomes and to avoid mixing count‐based and continuous‐expression statistical assumptions. For tuberculosis and preeclampsia, which were provided as raw count matrices, we applied the R package DESeq2 using its default size‐factor normalization and group‐wise comparison between disease and normal samples. For pancreatic ductal adenocarcinoma, where cfRNA data were provided in TPM format, we used the R package limma on log2‐transformed values. In all cases, genes with adjusted *p* value < 0.05 were considered differentially expressed. To obtain a robust set of disease‐associated markers, DE gene lists were subsequently merged with the cfRNA biomarkers identified from SHAP‐based interpretability analysis of the classification models. The merged cfRNA set served as the input features for subsequent deconvolution analysis.

### 4.4 Graph‐Regularized Matrix Factorization

Graph‐Regularized Matrix Factorization was originally proposed by Cai et al. and has since been widely adopted in bioinformatics, with applications ranging from metabolite‐disease association [42] and circRNA–disease association identification [43] to snoRNA–disease association identification [44], and so on. In this study, we adapted graph‐regularized nonnegative matrix factorization to integrate cfRNA transcriptomic data with single‐cell references, thereby inferring a sample–cell matrix and reconstructing sample‐specific single‐cell transcriptomic profiles. The optimization objective is defined as:

(1) $$\min_{U,V}∥X-U^{T}V∥+\alpha ∥U^{T}V∥+\beta ∥U^{T}F_{sample}U∥$$

where $X\in R^{r\times c}$ denotes input matrix. $U\in R^{s\times r}$ is basis matrix. $V\in R^{s\times c}$ is the coefficient matrix. α and β are regularization coefficients. In our matrix factorization setup, the input matrix *X* is initialized as the single‐cell reference matrix, the basis matrix *U* is initialized as the cfRNA expression matrix, and the coefficient matrix *V* is randomly initialized using NumPy's `*np.random.rand*` function, and the regularization coefficients α and β are initialized to 0.001 [43, 44]. *F_sample_* = *I_sample_*  − *S_sample_* represents the graph laplacian matrices for sample similarity matrix. *S_sample_* denotes the sample similarity matrix, which is computed from cfRNA transcriptomic data using cosine similarity. *I_sample_* is a diagonal matrix; the elements in *I_sample_* are row sums of *S_sample_*. *r*, *c*, and *s* represent the number of cfRNAs, cells, and samples, respectively. The first term minimizes the reconstruction error between the single‐cell reference matrix *X* and its low‐rank approximation *U^T^V*. The second term constrains the factorization by penalizing the complexity of *U^T^V*, thereby reducing the risk of overfitting. The third term incorporates graph regularization through the Laplacian matrix *F_sample_*, which preserves the intrinsic similarity structure among samples as defined by the cfRNA‐based cosine similarity network.

Based on Lagrange multipliers and Karush–Kuhn–Tucker (KKT) conditions [45], the Lagrange function corresponding to Equation (1) is formulated as:

(2) $$\begin{array}{ccc} \Gamma & = & \;\mathrm{Tr}\left(XX^{T}\right)-2\mathrm{Tr}\left(UXV^{T}\right)+\left(1+\alpha \right)\mathrm{Tr}\left(V^{T}UU^{T}V\right)\\ & & +\beta \mathrm{Tr}\left(U^{T}F_{sample}U\right)+Tr\left(\Lambda_{1}U^{T}\right)+Tr\left(\Lambda_{2}V\right) \end{array}$$

where Λ_1_ and Λ_2_ denote Lagrange multiplier. The partial derivatives of Equation (2) with respect to *U* and *V* are given by:

(3) $$\left\{\begin{array}{c} \frac{\partial \Gamma }{\partial U}=-2VX^{T}+2\left(1+\alpha \right)VV^{T}U+2\beta I_{sample}U-2\beta S_{sample}U+\Lambda_{1}\\ \frac{\partial \Gamma }{\partial V}=-2UX+2\left(1+\alpha \right)UU^{T}+\Lambda_{2}\;\;\;\;\;\;\;\;\;\;\;\;\;\;\;\;\;\;\;\;\;\;\;\;\;\;\;\;\;\;\;\;\;\;\;\;\;\;\;\;\;\;\;\;\;\;\;\;\;\;\;\;\;\;\;\;\;\;\;\; \end{array}\right.$$

Based on KKT conditions [45], the updating rules for *U* and *V* are as follow:

(4) $$\left\{\begin{array}{c} \;U_{ij}^{new}=U_{ij}\;\frac{VX^{T}+\beta S_{sample}U}{\left(1+\alpha \right)VV^{T}U+\beta I_{sample}U}\\ \;V_{ij}^{new}=V_{ij}\;\frac{UX}{\left(1+\alpha \right)UU^{T}}\; \end{array}\right.$$

non‐negativity is enforced by the multiplicative update rules (Equation 4), which preserves non‐negativity (≥0) given nonnegative initialization. We terminate the optimization based on two convergence criteria: (1) the algorithm stops once the maximum number of iterations is reached; and (2) it stops early if the relative change in the input matrix between two consecutive iterations becomes sufficiently small. Specifically, we compute *dA*  =  *X* − *X^t^*, where *X* is the original input matrix and *X^t^* is the reconstructed matrix at iteration *t*, and evaluate the normalized mean absolute difference *mean*(*dA*)/*mean*(*X*).

Finally, the predicted single‐cell matrix can be defined as *X_new_* = (*U^new^*)^*T*^ *V_new_*. This inferred matrix *X_new_* was subsequently used for cell–cell communication analysis to uncover disease‐specific intercellular signaling alterations. In addition, the sample–cell matrix *V_new_* quantified the associations between disease or normal samples and specific cell types, thereby enabling both disease stratification and the tracing of cellular origins.

### 4.5 Cell–Cell Communication Analysis

Cell–cell communication analysis was performed using the CellChat R package [46]. The predicted single‐cell transcriptomic profiles *X_new_* inferred by CellFreeGMF for disease and normal groups were used as input. To harmonize gene identifiers, Ensembl IDs were mapped to official gene symbols using *org.Hs.eg.db*. Genes without valid symbols were removed. Duplicated gene symbols were deduplicated to ensure one‐to‐one mapping. Disease and normal datasets were processed separately. CellChat objects were created using *createCellChat* with cell‐type annotations provided in the metadata and *group.by = “cell_type”*. For ligand–receptor prior knowledge, we used the human CellChat database [46]. Prior to inferring communication probabilities, genes were subset to those present in the corresponding expression matrices using *subsetData*. Overexpressed genes and interactions were then identified using *identifyOverExpressedGenes* and *identifyOverExpressedInteractions*. Protein–protein interaction information was incorporated by projecting onto the human PPI network using *projectData* with *PPI.human*. Communication probabilities were computed with *computeCommunProb(raw.use = TRUE, population.size = TRUE)*. Inferred interactions were filtered to retain only cell groups with at least 3 cells using *filterCommunication(min.cells = 3)*. Pathway‐level communication and aggregated networks were obtained using *computeCommunProbPathway* and *aggregateNet*, respectively. Network centrality metrics were computed with *netAnalysis_computeCentrality (slot = “netP”)*. To quantify global communication patterns, we applied the “*compareInteractions”* function. The differential interaction networks were visualized using “*netVisual_diffInteraction”* function and summarized by *“netVisual_heatmap”* function. To explore cell‐type–specific differences, we generated bubble plots with the *“netVisual_bubble”* function. These analyses enabled the identification of significantly increased or decreased ligand–receptor interactions, as well as differentially regulated signaling pathways between disease and normal groups.

## Funding

This research was funded by the Basic Science Center Program of National Natural Science Foundation of China (T2288102), National Key R&D Program of China, MOST (2023YFC2509900), National Key R&D Program Priority Special Projects of China (2024YFC3016605), National Natural Science Foundation of China (32371048), Guangdong Medical Research Fund (B2025088), Beijing Municipal Natural Science Foundation – Fengtai Joint Fund (2024FTQY037), Shenzhen Medical Research Fund (D2401015), Shenzhen Clinical Research Center for Trauma Treatment (20230731111952004).

## Conflicts of Interest

The authors declare no conflicts of interest.

## Supporting information




**Supporting File 1**: advs74814‐sup‐0001‐SuppMat.docx.

**Supporting File 2**: advs74814‐sup‐0002‐TableS1‐S4.xlsx.

## Data Availability

The authors analyzed three publicly available cfRNA clinical datasets. The data were acquired from the following accession numbers: (1) tuberculosis data (GSE255071, GSE255073, and GSE255074) [6]; (2) preeclampsia data (GSE192902) [7]; (3) pancreatic ductal adenocarcinoma data (GSE133684) [11]. Pancreatic adenocarcinoma omics data were downloaded from the TCGA portal (https://portal.gdc.cancer.gov). All data supporting this study are included in the article and Supplementary Information, or can be obtained from the authors upon reasonable request. The CellFreeGMF code and Supplementary Table are openly available on GitHub (https://github.com/zwx94/CellFreeGMF) and can be installed via either PyPI (https://pypi.org/project/CellFreeGMF/) or Anaconda. Comprehensive tutorials for installation and usage are provided at https://cellfreegmf.readthedocs.io/. The package is implemented in Python, with version 3.10.16 recommended.
