# Supplementary material for: From Cell‐Free Transcriptomes to Single‐Cell Landscapes: Biomarker Discovery and Originating Cell Alteration Analysis via Graph Matrix Factorization
Source: Adv Sci (Weinh). 2026 Mar 12;13(29):e74814. doi: 10.1002/advs.74814 (PMC13205738; doi:10.1002/advs.74814)
Supplement: Supplementary file 1 — Supporting File 1: advs74814‐sup‐0001‐SuppMat.docx. [file ADVS-13-e74814-s002.docx]

**Supplementary Information for “From Cell-Free Transcriptomes to Single-Cell Landscapes: Biomarker Discovery and Originating Cell Alteration Analysis via Graph Matrix Factorization**”

***Wenxiang Zhang^1,2,3,#^, Wenjing Zhang^1,2,3,#^, Hang Wei^4^, Shiyan Liu^1^, Junliang Shang^5^, Weijie Gong^6^, Hanwen Cheng^3,8^, Xiujuan Lei^7*^, Yuhui Kou^3,8,*^, Baoguo Jiang^1,3,8,*^***

### **Affiliations**

^1^ Shenzhen Clinical Research Center for Trauma treatment, Shenzhen University General Hospital, Shenzhen University, Shenzhen, 518055, China

^2^ Guangdong Key Laboratory for Biomedical Measurements and Ultrasound Imaging, National-Regional Key Technology Engineering Laboratory for Medical Ultrasound, School of Biomedical Engineering, Shenzhen University Medical School, Shenzhen 518060, China

^3^ National Center for Trauma Medicine, Beijing 100000, China

^4^ School of Computer Science and Technology, Xidian University, Xi’an, Shaanxi 710126, China;

^5^ School of Computer Science, Qufu Normal University, Rizhao, 276826, China

^6^ Department of Family Medicine, Shenzhen University Medical School, Shenzhen, Guangdong 518060, China

^7^ School of Artificial Intelligence and Computer Science, Shaanxi Normal University, Xi’an 710119, China

^8^ Department of Orthopedics and Trauma, Peking University People's Hospital, Beijing, 100044, China

* Corresponding authors:

Correspondence and requests for materials should be addressed to Yuhui Kou (yuhuikou@bjmu.edu.cn).


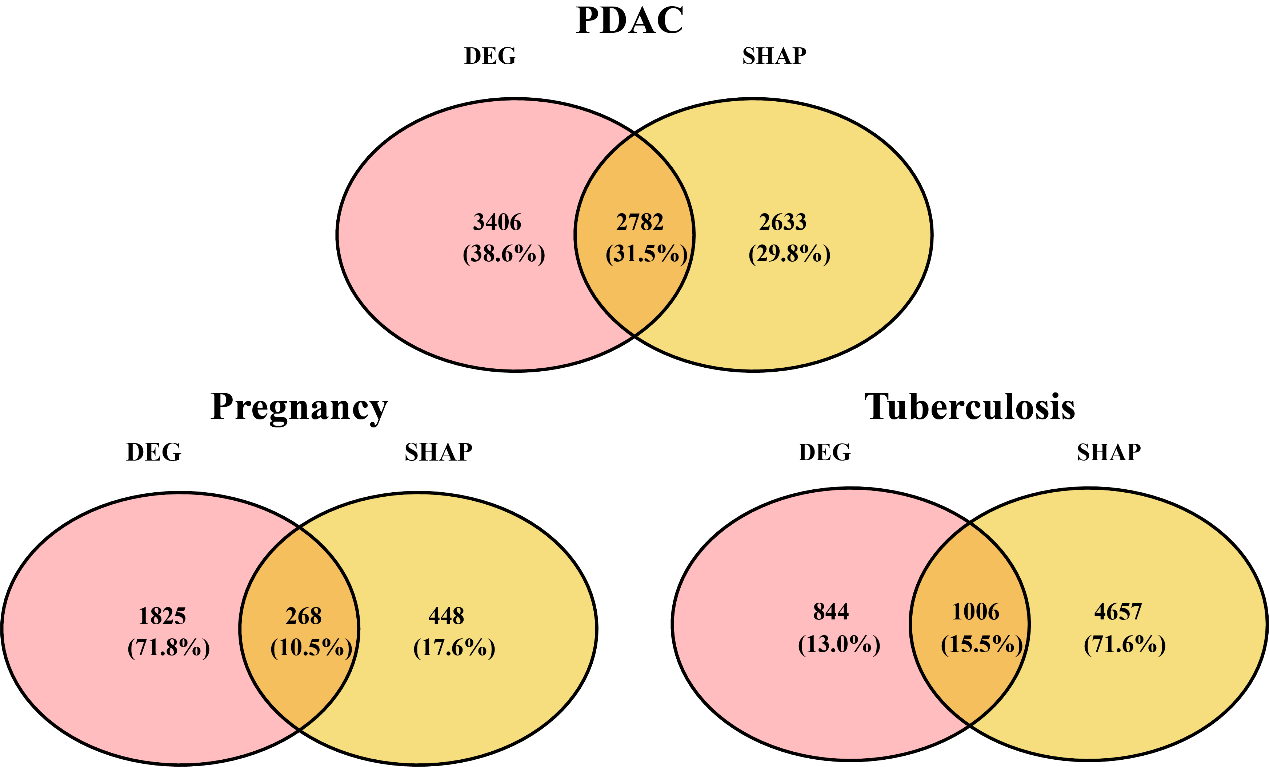


**Figure S1** | **Venn diagram showing the overlaps and differences of cfRNA biomarkers identified by different methods.**


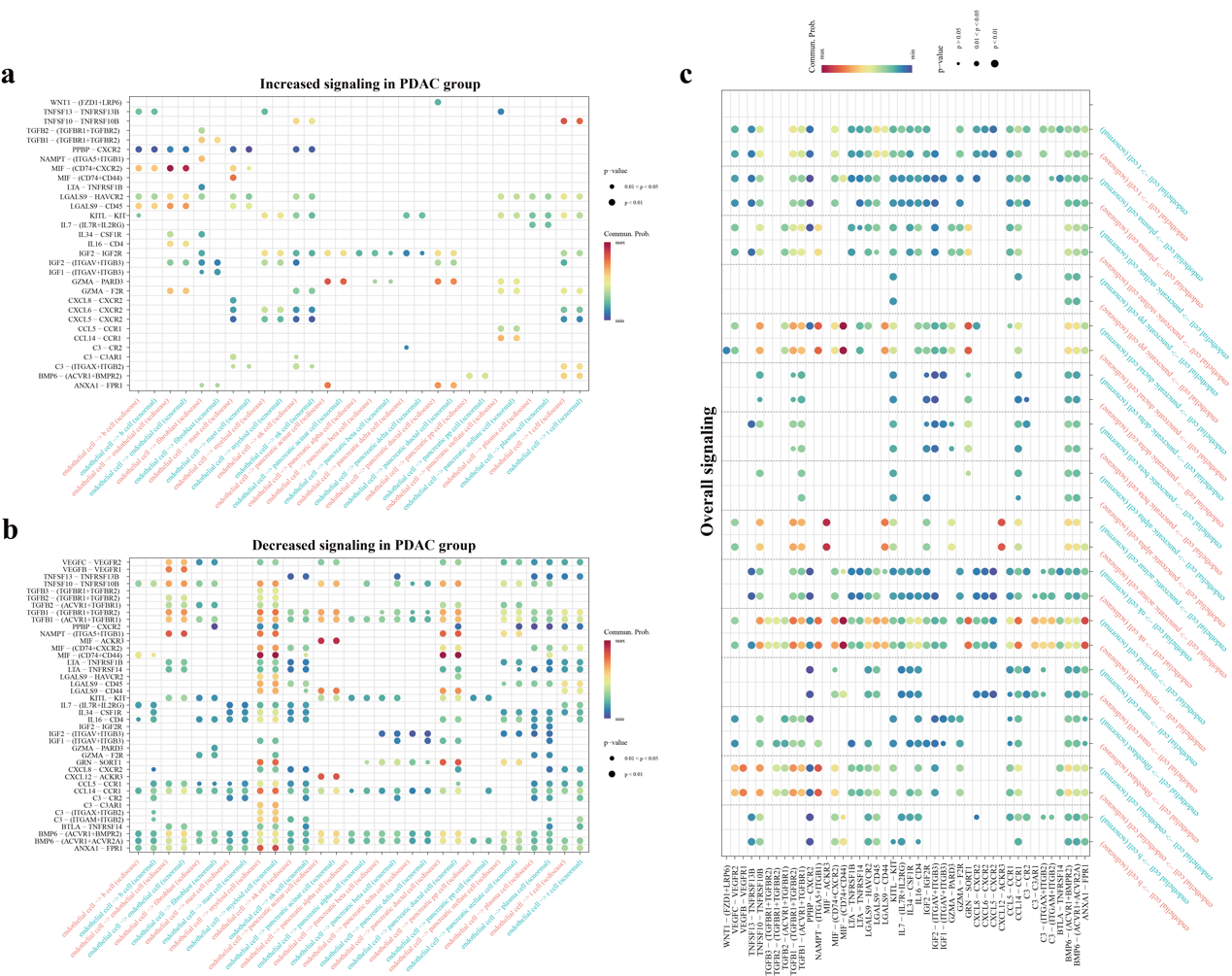


**Figure S2 | Bubble plots showing significantly difference interactions in the PDAC group compared with the normal group for endothelial cell.**


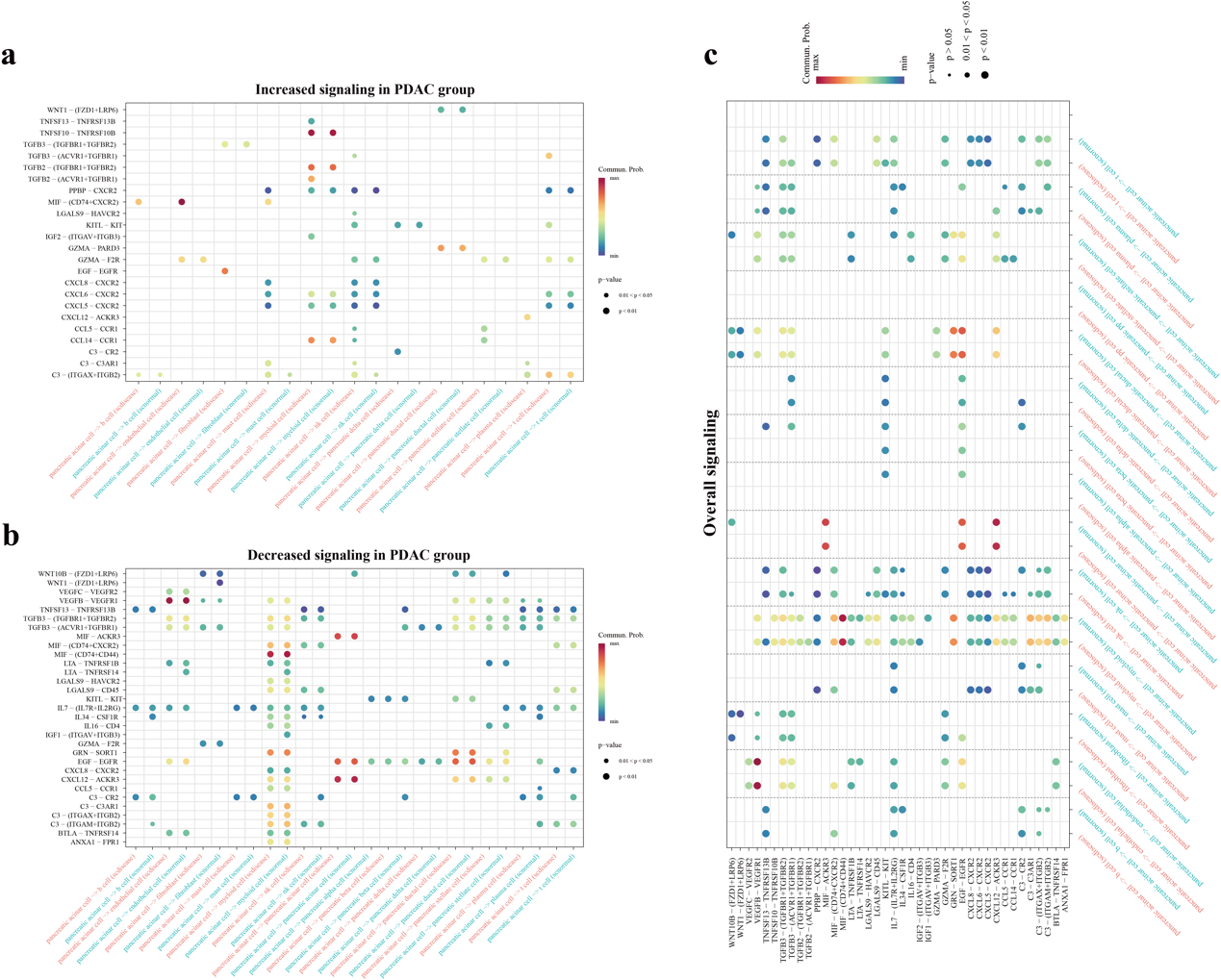


**Figure S3 | Bubble plots showing significantly difference interactions in the PDAC group compared with the normal group for pancreatic acinar cell.**


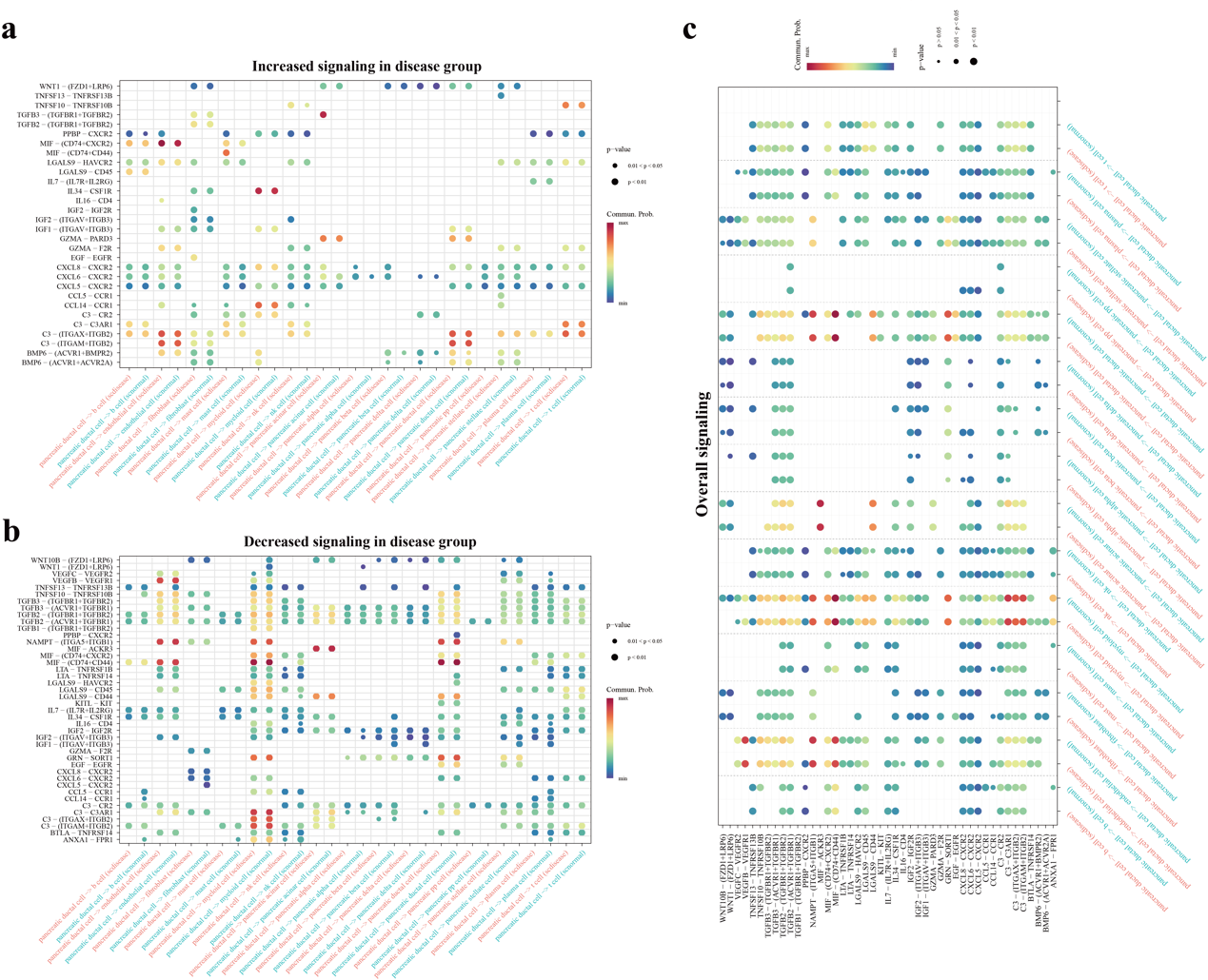


**Figure S4 | Bubble plots showing significantly difference interactions in the PDAC group compared with the normal group for pancreatic ductal cell.**

**Figure S5** | **Pathway-level differential analysis.** **a**, Functional similarity map of signaling pathways into two-dimensions space. **b**, The signaling networks exhibit greater or smaller differences based on their Euclidean distances in the shared two-dimensional space. A larger distance indicates a greater dissimilarity between the communication networks of two datasets. **c**, Significant signaling pathways were ranked based on differences in the overall information flow within the inferred networks between two datasets. The top signaling pathways colored red are enriched in PDAC group, and these colored greens were enriched in the normal group.


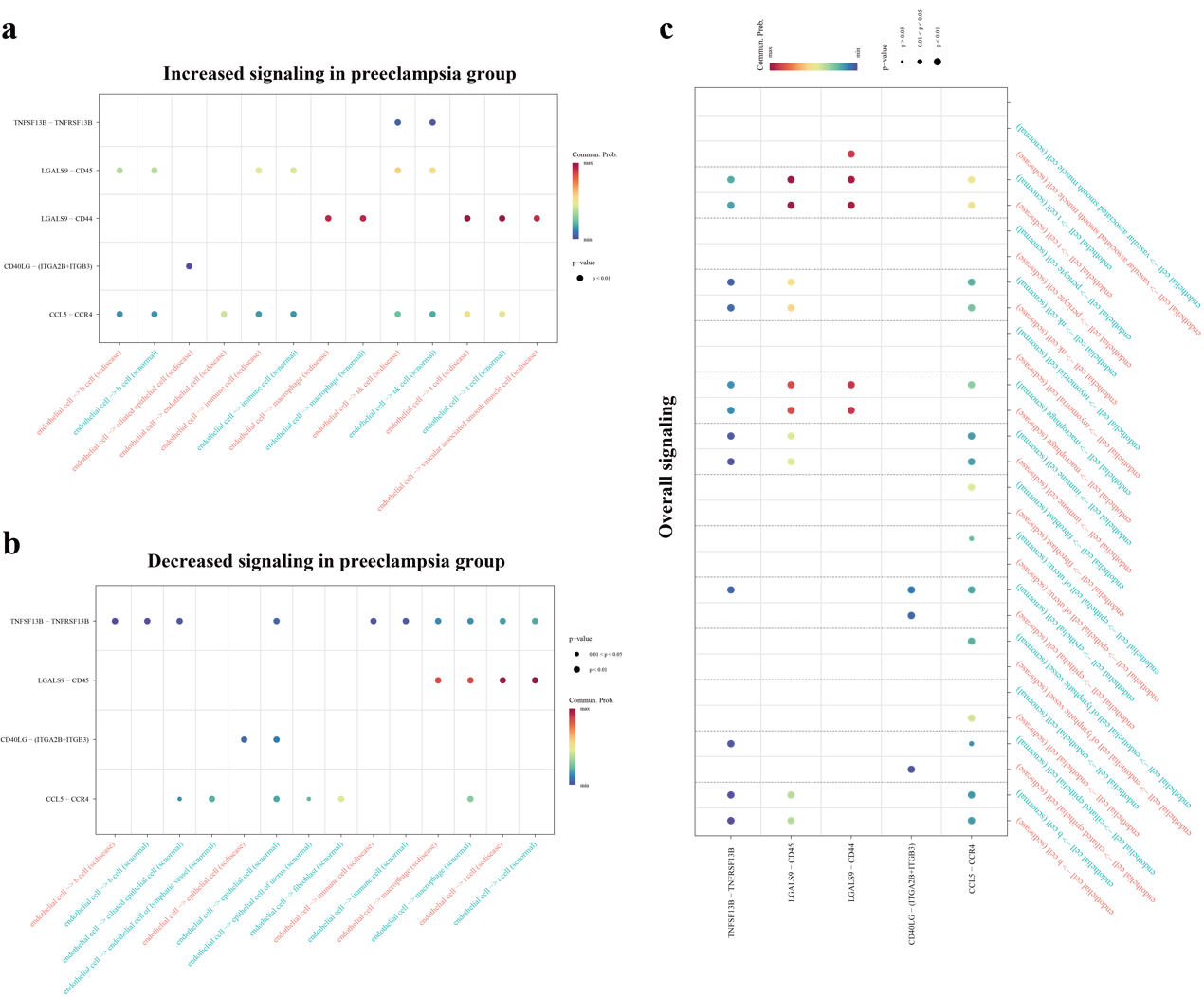


**Figure S6** | **Bubble plots on endothelial cell showing significantly difference interactions in the preeclampsia group compared with the normal group.**


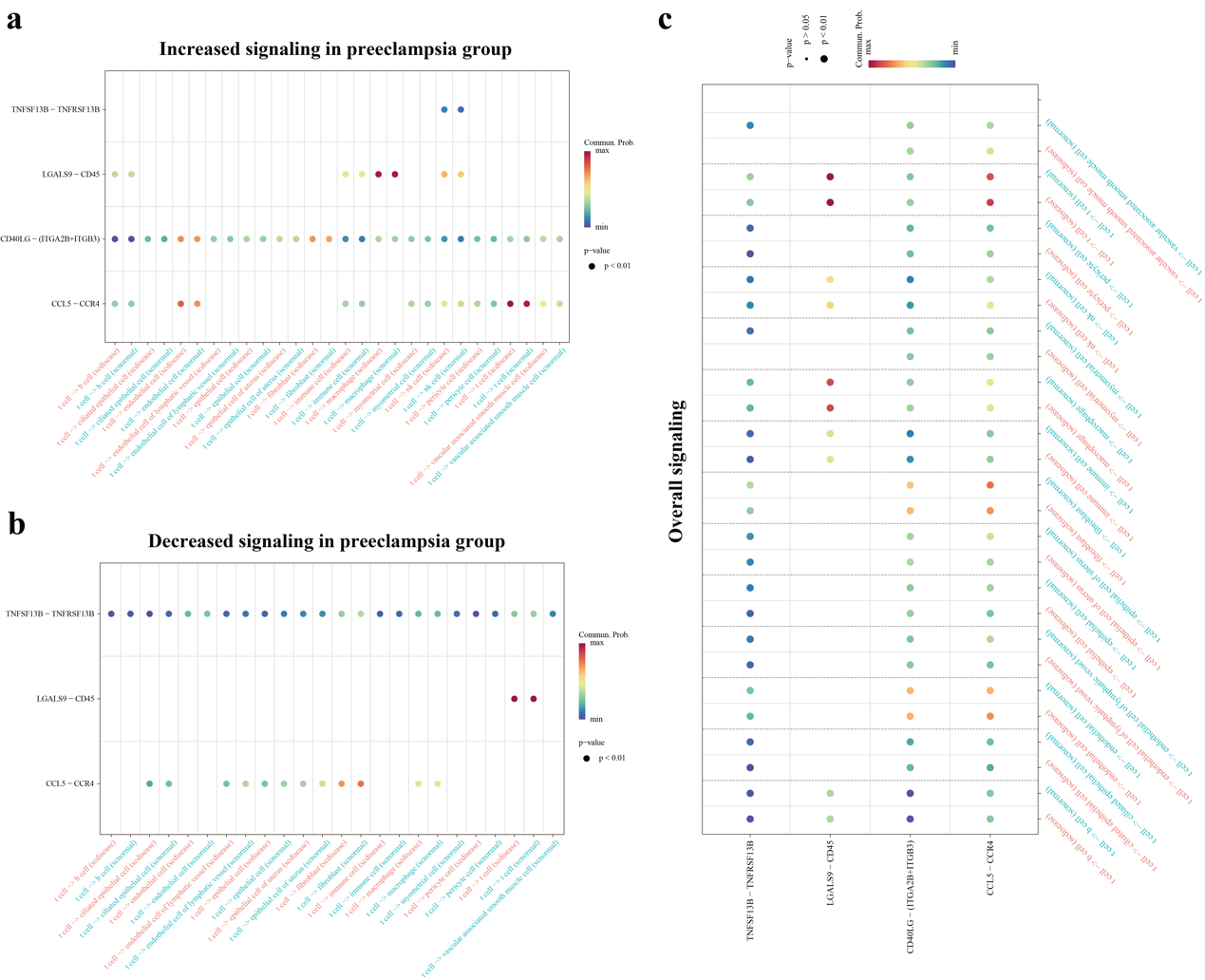


**Figure S7** | **Bubble plots on t cell showing significantly difference interactions in the preeclampsia group compared with the normal group.**


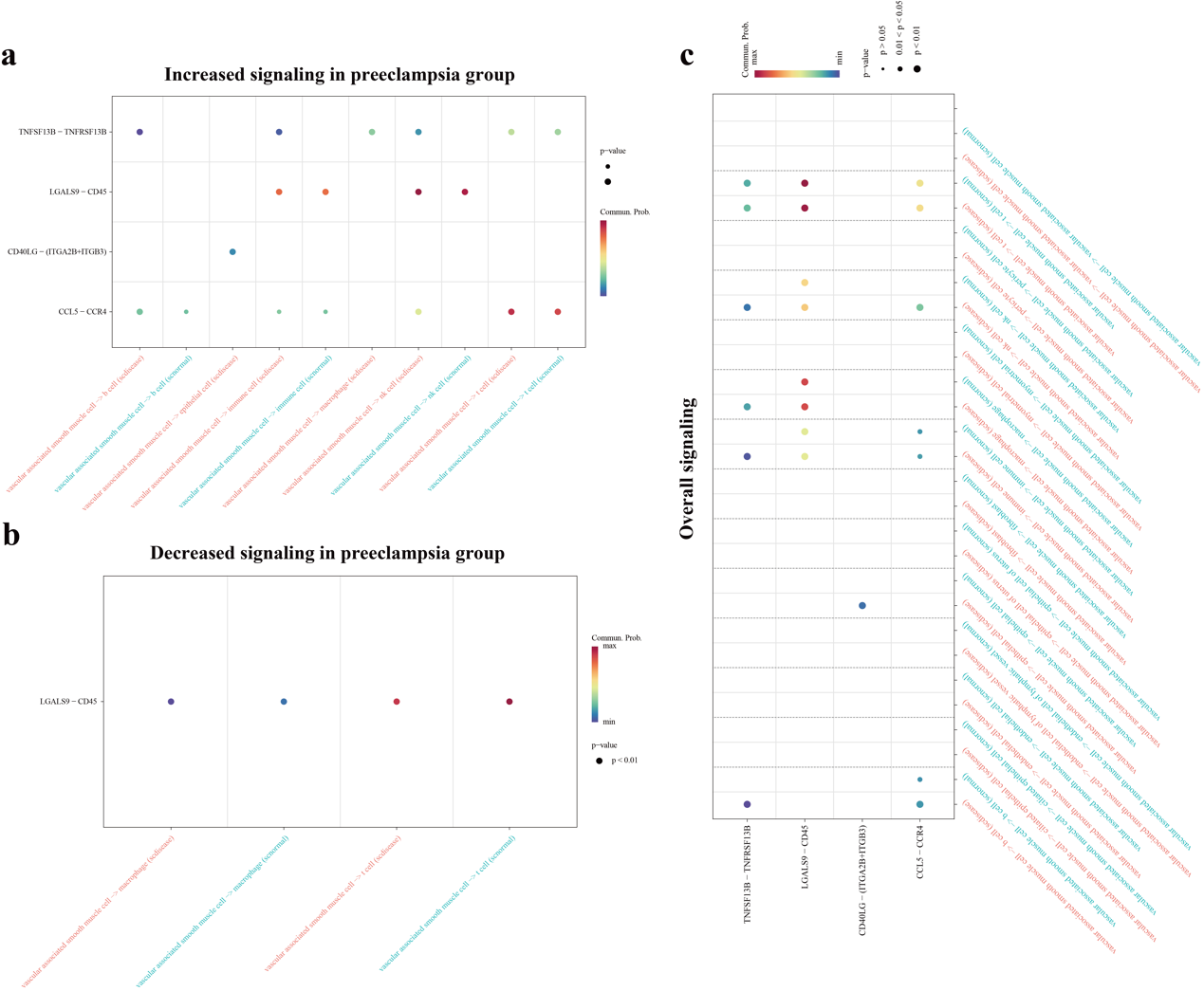


**Figure S8** | **Bubble plots on vascular associated smooth muscle cell showing significantly difference interactions in the preeclampsia group compared with the normal group.**


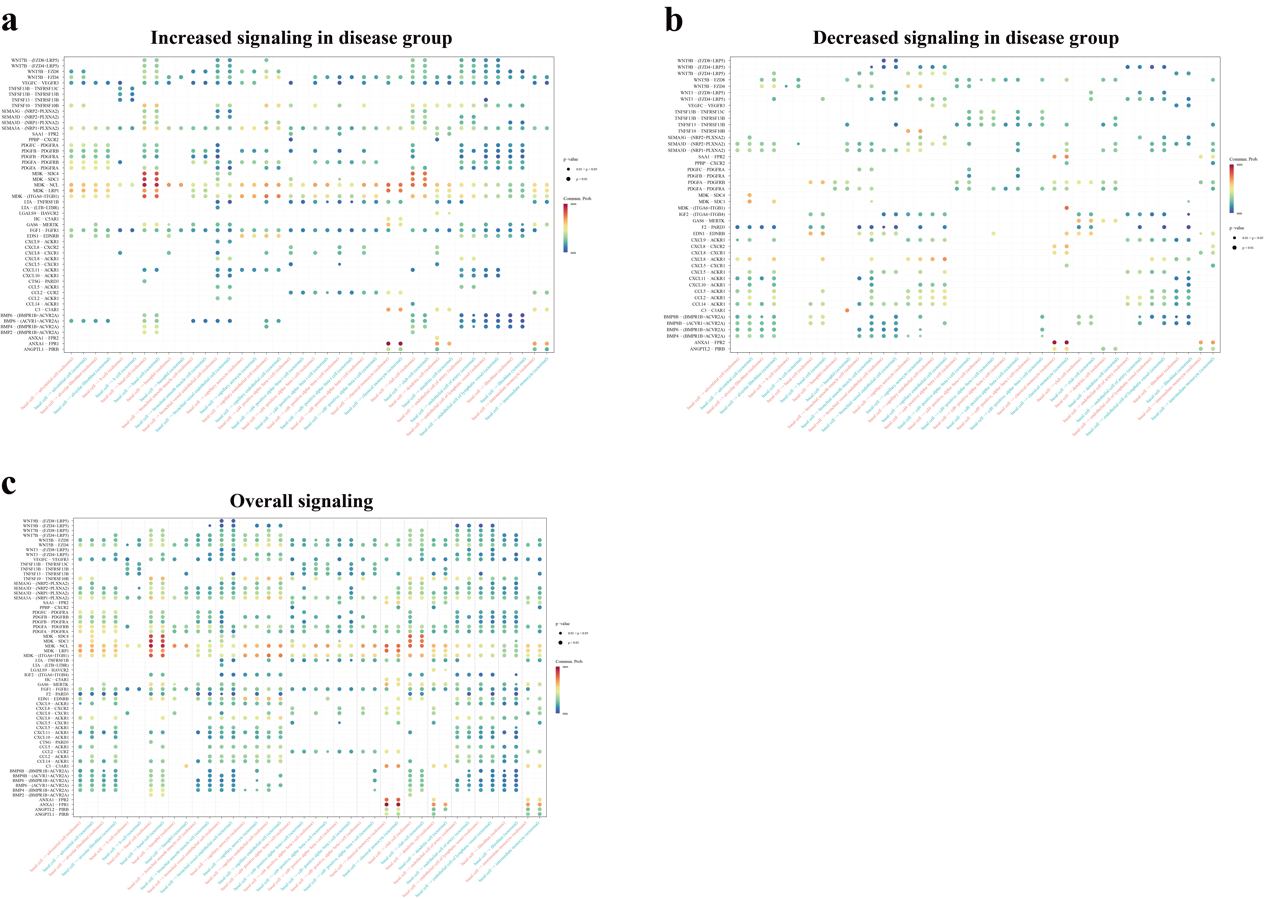


**Figure S9** | **Bubble plots on basal cell showing significantly difference interactions in the tuberculosis group compared with the normal group.**


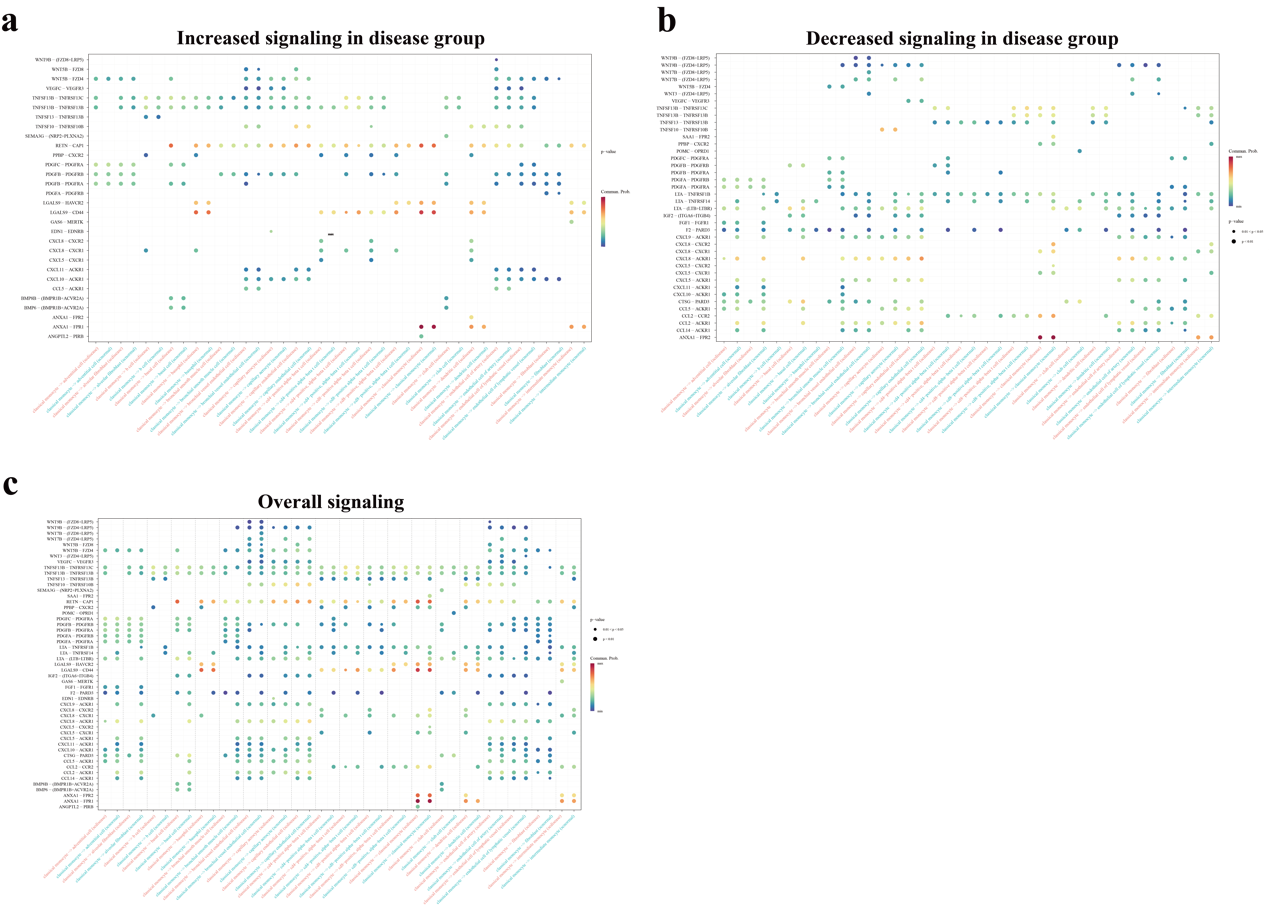


**Figure S10** | **Bubble plots on classical monocyte showing significantly difference interactions in the tuberculosis group compared with the normal group.**


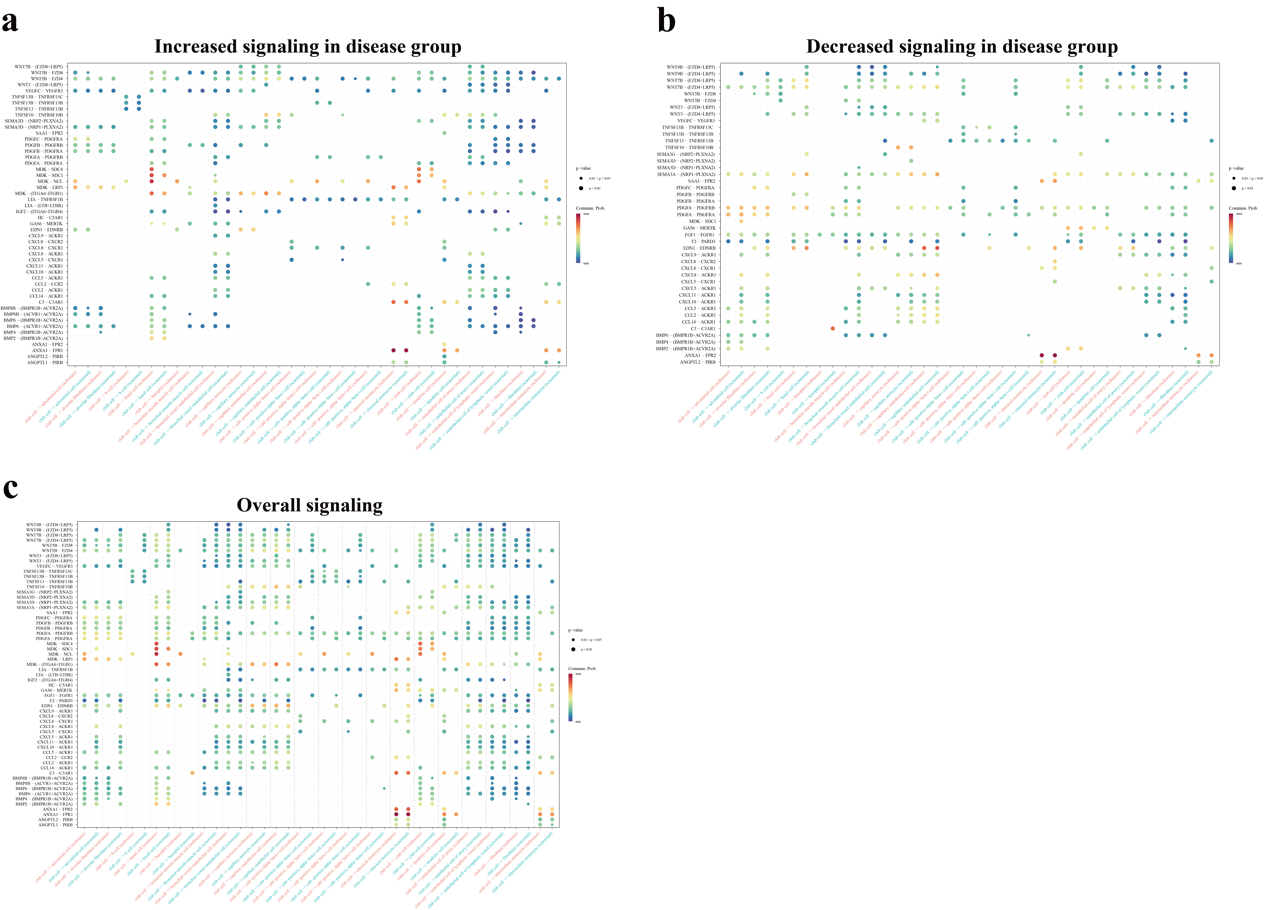


**Figure S11** | **Bubble plots on club cell showing significantly difference interactions in the tuberculosis group compared with the normal group.**


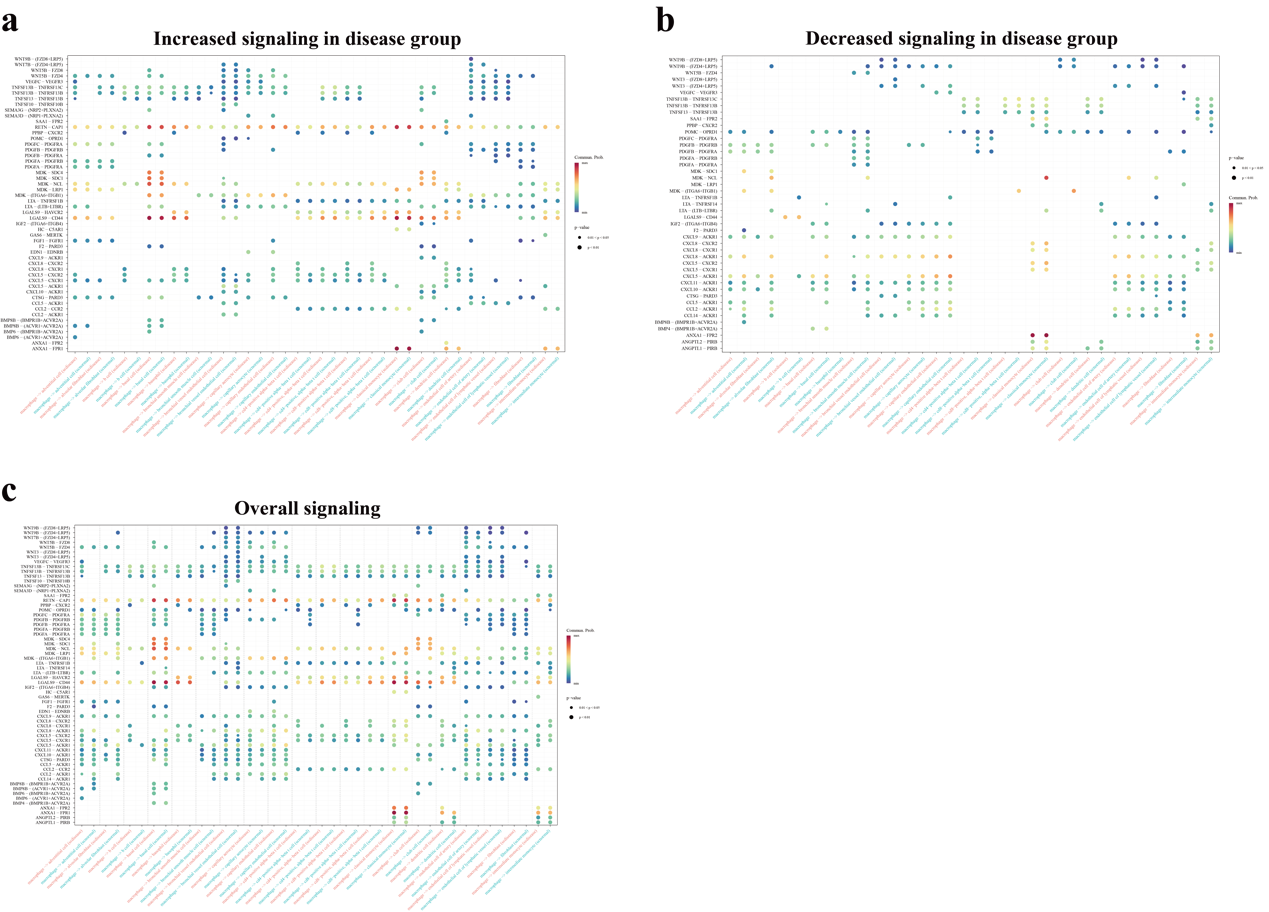


**Figure S12** | **Bubble plots on macrophage cell showing significantly difference interactions in the tuberculosis group compared with the normal group.**
